# Educational

**Published:** 1896-12

**Authors:** 


### EDUCATIONAL.

COLORADO will be among the States that will ask in behalf of her veterinary profession legislation looking to their proper recognition. They are organized for the battle, and will earnestly press for success. A draft of a bill has been approved by the Colorado Veterinary Association at a recent meeting.

Live-stock judging is taught at the Wisconsin Agricultural College at Madison.

The relative value of the meat of open heifers, spayed heifers, and steers is the subject of an interesting bulletin from the Iowa Experimental Station, and the injustice of price done heifer-meat in the market fully exposed.

The Ohio Dairy School at the State University has as a portion of the course breeds, breeding, feeding, and diseases of dairy-cattle.

A leading periodical of Berlin, Germany, denies that it is the intention to prevent the importation of canned meats.

The new building for the department of veterinary surgery of the Detroit College of Medicine will be completed in two weeks. When the building is finished and the department removed from the old building on Beaubine Street, where now located, the students of the veterinary department will be as well provided for the study of diseases of the domesticated animals as the students of the medical department are now provided with the means of studying the diseases of the human family. The new building is back of the Detroit Medical College building on Mullet Street, a handsome four-story structure with graystone finishing. The entrance is on Mullet Street between a pair of graystone pillars, and leads by a flight of stairs to the first floor above the basement. Upon the left-hand side of the long hallway that traverses the building longitudinally is the humane agent's office. Upon the right side is the office of the College of Veterinary Surgery. Upon this same floor are bed-rooms for those in charge of the reading-room and library, a private laboratory, and lecture-room. The second floor is to be used for laboratories and the other departments of the college. To the right of the building a wide driveway leads down to the basement entrance. The driveway is so arranged that the horse-ambulance can be driven right through the basement of the wing that projects to the right of the main building. In this wing are located the operating- and dissecting-rooms of the veterinary college. In the operating-room is a forge that will be used in demonstrating practical horseshoeing to the class; also all the necessary appliances required in operative surgery. The infirmary is finished in hardwood, with cement floor; hot and cold water, steam-heat, and electric lights.

Dr. Robert Ward, of Baltimore, late State Veterinarian of Maryland, proposes to give a series of lectures in November and December on "The Structural Anatomy and Conformation of the Various Breeds of Horses and their General Management in Health and Disease," and a special course for horse-shoers, treating on the foot. Fee for the course, ten dollars.

